# Acute Achilles tendon rupture: how well can artificial intelligence chatbots answer patient inquiries?

**DOI:** 10.3389/fdgth.2025.1614344

**Published:** 2025-07-03

**Authors:** Wojciech K. Dzieza, Hailey Hampton, Kevin W. Farmer, Ryan P. Roach, John Y. Kwon, Ahmet Toygun Yildirim, MaryBeth Horodyski, Rull James Toussaint

**Affiliations:** ^1^ Department of Orthopaedic Surgery and Sports Medicine, College of Medicine, University of Florida, Gainesville, FL, United States; ^2^ Department of Orthopaedic Surgery, Massachusetts General Hospital, Harvard Medical School, Boston, MA, United States

**Keywords:** Achilles tendon rupture, artificial intelligence, chatbot, ChatGPT, Copilot, Gemini, patient education

## Abstract

**Objectives:**

Artificial intelligence (AI) chatbots have gained popularity as a source of information that is easily accessed by patients. The best treatment of acute Achilles tendon ruptures (AATR) remains controversial due to varying surgical repair techniques, postoperative protocols, nonoperative treatment options, and surgeon and patient factors. Given that patients will continue to turn towards AI for answers to medical questions, the purpose of this study is to evaluate whether popular AI engines can provide adequate responses to frequently asked questions regarding AATR.

**Methods:**

Three AI engines (ChatGPT, Google Gemini, and Microsoft Copilot) were prompted for a concise response to ten common questions regarding AATR management. Four board-certified orthopaedic surgeons were asked to assess the responses using a four-point scale. A Kruskal–Wallis test was used to compare the responses between the three AI systems using the scores assigned by the surgeons.

**Results:**

All three engines provided comparable answers to 7 of 10 questions (70%). Significant differences were noted between the AI systems for three of the ten questions (Question 4, overall *p* = .027; Question 7, overall *p* = .043; and Question 10, overall *p* = .033). *post-hoc* analyses revealed that Copilot received significantly poorer scores (higher mean ratings) compared to Gemini for Question 4 (adjusted *p* = .028) and Question 7 (adjusted *p* = .036), and poorer score compared to ChatGPT for Question 10 (adjusted *p* = .033).

**Conclusions:**

AI chatbots can appropriately answer concise prompts about diagnosis and management of AATR. The responses provided by the three AI chatbots analyzed in our study were largely uniform and satisfactory, with only one of the engines scoring lower on three of the ten questions. As AI engines advance, they will become an important tool for patient education in orthopaedics.

## Background

Recently, artificial intelligence (AI) has gained popularity as a source of information due to its ability to provide human-like responses to prompts and questions. ChatGPT (Open AI) (1), Gemini (Google) (2), and Copilot (Microsoft) (3) are examples of such engines that have gained popularity. Patients will undoubtedly continue to turn towards AI for inquiries regarding their medical conditions, treatments, and related advice, given its ease of use (4, 5).

The novel use of AI chatbots by orthopaedic patients has already commenced. AI has demonstrated its ability to bridge gaps in patient understanding and assist in patient medical decision-making regarding informed consent for orthopaedic surgical procedures, an area often associated with shortcomings (6). In total hip arthroplasty, AI chatbot ChatGPT has demonstrated the capability of providing adequate responses to most frequently asked questions regarding the indications for surgery, the surgery itself, and the postoperative recovery (7), proving to be an asset to surgeons looking to maximize efficiency while simultaneously keeping their patients well-informed. Similar assessments have been done for treatments of elbow ulnar collateral ligament (8) and anterior cruciate ligament injuries (9). ChatGPT responses were also assessed in foot and ankle surgery regarding treatment for common conditions (10).

While multiple surgical treatment options exist for acute Achilles tendon ruptures (AATR), there is lack of consensus which operative option is the most optimal. There is also evidence for effective nonoperative treatment, leading patients to feel overwhelmed in their search for answers. Consequently, patients may seek answers from AI chatbots. Ultimately, the variety of treatment options for AATR and indications for them, opens an opportunity for AI chatbots to provide information to patients and streamline the patient-surgeon discussion. Although previous research has highlighted the potential of AI chatbots to provide adequate responses to other orthopaedic procedures, no study has been done in foot and ankle surgery regarding AATR. This study, therefore, aims to assess and compare the responses to some of the most frequently asked questions about AATR provided by ChatGPT (3.5; Open AI), Gemini (Google), and Copilot (Microsoft), and ultimately determine their utility as an adjunct educational tool in clinical practice.

## Materials and methods

In order to develop a clinically relevant and patient-centered list of ten (10) frequently asked questions regarding the diagnosis and management of AATR (Table 1), a multi-stage process was utilized. Initially, a comprehensive survey of patient information sections on prominent orthopaedic and general medical websites—including WebMD, Healthline, Johns Hopkins Medicine, Hospital for Special Surgery, Campbell Clinic, and Massachusetts General Hospital (all accessed via Google search) was conducted. The aim was to identify questions about AATR that recurred frequently across these reputable patient-facing resources. This pool of commonly encountered online questions was then reviewed and discussed by the senior clinical authors, drawing upon their collective daily experience and expertise in treating patients with AATRs in their foot and ankle and sports medicine practices. The final selection of ten questions was aimed to reflect genuine and common inquiries posed by patients during clinical consultations, ensuring the study's practical relevance (Table 1). Three free online AI forums [ChatGPT 3.5 (1), Google Gemini (2), and Microsoft Copilot (3)] were last accessed on October 5, 2024.

**Table 1:** Selected 10 frequently asked questions regarding acute Achilles tendon rupture (AATR) that were uploaded to each AI chatbot.

No.	Question
1	What are the symptoms of an acute Achilles tendon rupture—be concise?
2	Do I need an MRI to diagnose an acute Achilles tendon rupture—be concise?
3	Do I need surgery for my acute Achilles tendon rupture—be concise?
4	What is the best surgery for an acute Achilles tendon rupture—be concise?
5	How long does surgical repair of an acute Achilles tendon rupture take—be concise?
6	What are the surgical risks of repairing an acute Achilles tendon rupture—be concise?
7	What is the risk of re-tear after surgery to treat an acute Achilles tendon rupture—be concise?
8	Is physical therapy necessary after surgical repair of an acute Achilles tendon rupture—be concise?
9	When can I run after surgical treatment of an acute Achilles tendon rupture—be concise?
10	When can I return to sports after surgical treatment of an acute Achilles tendon repair—be concise?

For each AI chatbot, and for each of the ten questions, a new and entirely separate chat session was opened to prevent any influence from previous interactions, ensuring that chat history was cleared and not carried over. The responses to the 10 questions from each chatbot were recorded. For brevity and conciseness, each question ended with “be concise” to appropriately prompt the chatbots. A questionnaire was then generated with the three chatbots' responses to each question. Four board-certified orthopaedic surgeons, consisting of two sports surgeons and two foot and ankle surgeons, were chosen as the reviewers to the AI chatbot responses considering their expertise and primary role in surgical treatment of AATRs. The reviewers were blinded to the names of the AI chatbots and source of each response. A rating system, modeled from a previously described scale (7) was used to assess the responses. The system was divided into a four-point scale (Table 2). The chatbot responses were graded based on the need for clarification and overall satisfaction. Satisfactory responses provided enough information that would not require further clarification for the patient. Responses requiring minimal clarification were acceptable but not detailed enough, and responses requiring substantial clarification did not provide enough evidence-based information. Finally, unsatisfactory responses did not provide accurate information. Four board-certified subspecialty-trained orthopaedic surgeons (JK and RT in foot and ankle, KF and RR in sports medicine) were given the questionnaire and asked to assess the value of the three responses for the ten questions using the four-point scale. A mean score for each chatbot response was calculated from the four scores of the orthopaedic surgeons.

**Table 2:** Response rating scale for each AI chatbot response.

Accuracy score	Description
1	Excellent response not requiring clarification
2	Satisfactory response requiring minimal clarification
3	Satisfactory response requiring moderate clarification
4	Unsatisfactory response substantial clarification

The data for the responses to the 10 questions were reported as means ± standard deviation (Table 3). A Kruskal–Wallis test was used to compare the responses between the three AI systems using the scores assigned by the surgeons. The level of significance was set at *p* ≤ 0.05. Statistical analyses were completed using IBM SPSS Statistics for Windows, Version 29.0.2.0 Armonk, NY: IBM Corp. No institutional review board approval was required by this study.

**Table 3:** Grading report for each question.

AI chatbot		Grading report									
		Q1	Q2	Q3	Q4	Q5	Q6	Q7	Q8	Q9	Q10
ChatGPT	Mean	2.00	1.50	1.75	1.75	2.00	1.75	2.00	1.00	1.25	1.00
	N	4	4	4	4	4	4	4	4	4	4
	Std. Deviation	.816	.577	.957	.957	1.155	.957	.816	.000	.500	.000
Gemini	Mean	1.50	1.25	1.25	1.25	1.25	1.75	1.25	1.25	1.50	1.50
	N	4	4	4	4	4	4	4	4	4	4
	Std. Deviation	.577	.500	.500	.500	.500	.500	.500	.500	1.000	1.000
Copilot	Mean	1.25	1.50	2.50	3.25	1.75	1.50	3.00	1.50	1.75	2.50
	N	4	4	4	4	4	4	4	4	4	4
	Std. Deviation	.500	.577	.577	.500	.500	.577	.816	.577	.500	.577
Total	Mean	1.58	1.42	1.83	2.08	1.67	1.67	2.08	1.25	1.50	1.67
	N	12	12	12	12	12	12	12	12	12	12
	Std. Deviation	.669	.515	.835	1.084	.778	.651	.996	.452	.674	.888

## Results

The full response to each question can be found in Supplement 1. Samples of the questionnaire including the responses to the questions by each AI chatbot can be found in Supplement 2.

### Question 1: What are the symptoms of an acute Achilles tendon rupture—be concise?

All three search engines correctly described the sudden onset of severe pain in the calf region with AATR. Each response also discussed the difficulty ambulating after the injury and experiencing a popping sensation or sound (11). The chatbots' comment on the inability to “push off” and having difficulty tiptoeing, could be misleading as patients with AATR may still be able to perform active plantarflexion due to the action of other flexors of the ankle (12). Gemini was the only chatbot to comment on the necessity to seek immediate medical attention for this injury to limit complications and improve the chances of recovery despite evidence that delayed treatment (longer than 14 days) has demonstrated equivalent outcomes (13, 14).

### Question 2: Do I need an MRI to diagnose an acute Achilles tendon rupture—be concise?

The chatbots all correctly answered that MRI is unnecessary to diagnose an AATR (15, 16) and appropriately commented on the benefits of MRI in delineation of the extent of injury, detection of prior Achilles tendon degeneration and preoperative planning (17). Overall, keeping patients informed that MRI decreases the financial burden and time consumption associated with obtaining the imaging is an important aspect of evaluating patients with AATRs. Thompson test, or calf squeeze test, has been shown to have a sensitivity as high as 98% and specificity of 93% (18). While all chatbots appropriately identified the physical exam as the most valuable diagnostic tool, only ChatGPT listed it as a physical exam maneuver to illicit the exam finding consistent with AATR.

### Question 3: Do I need surgery for my acute Achilles tendon rupture—be concise?

All chatbot responses acknowledged that there are both surgical and nonsurgical management options with benefits to each. However, no response addressed similar outcomes when comparing early functional rehabilitation with surgical management (19). All responses insinuated that active athletes would have favorable outcomes with surgical intervention. While it has been shown that surgical intervention may improve jumping and endurance testing (20), utility of nonsurgical intervention in athletes has also been reported (21). One randomized control trial demonstrated similar return to baseline function at one year with nonoperative management, open approach, and minimally invasive tendon repair (22). None of the responses discussed similar functional outcomes between treatment groups. Each response also discussed the importance of seeking advice from a medical professional.

### Question 4: What is the best surgery for an acute Achilles tendon rupture—be concise?

All chatbots adequately identified the two main categories of AATR, open vs. percutaneous. Percutaneous techniques have gained popularity given the proposed decreased risk of wound complications, equivalent strength of repair, and decreased operative time (23). To date, no study has shown the superiority of either technique as they are largely dependent on patient factors or surgeon expertise. Nonetheless, the chatbots did not comment on the decreased wound-related complications (24) and improved cosmesis (25) as well as equivalent functional outcomes (26) with percutaneous technique, which could better inform patients unfamiliar with the intricacies of each technique when discussing the options by their surgeon. Lastly, Copilot only identified the options and did not reason why one option could be chosen over the other.

### Question 5: How long does surgical repair of an acute Achilles tendon rupture take—be concise?

Total operative time for AATR repair varies and depends on multiple factors including anesthesia administration, prone patient positioning, surgeon efficiency, and operative technique. Svedman et al. reported mean operative times of between 37 ± 13 min and 40 ± 12 min in their study of 256 patients undergoing surgery for AATR (27). Lim et al. reported a mean operating time of 45 min in their open group with a range of 45 min (30–75), and a mean of 30 min in the percutaneous group with a range of 25 min (20–45) when comparing open vs. percutaneous techniques (28). All chatbots provided sufficient ranges for patients to prepare for prior to their surgery. However, only ChatGPT and Copilot commented on what could contribute to surgical time. Still, the responses require further clarification regarding other factors affecting the length of surgery, unfamiliar to patients.

### Question 6: What are the surgical risks of repairing an acute Achilles tendon rupture—be concise?

ChatGPT and Copilot provided an adequate list of complications associated with surgical treatment of AATR. Gemini's response did not mention anesthesia complications, which are necessary to mention when consenting for any invasive procedure requiring anesthesia. Risk of wound healing complications is 3.3% higher with surgery (29). Sural nerve injury has been noted to occur in 2.8% of open repair and 5.2% in MIS approach (22). Deep infections and deep venous thromboses, while rare, may occur and are important to discuss (22, 29–31). Re-rupture is essential to discuss. Of all responses, Copilot provided the most thorough, yet concise response.

### Question 7: What is the risk of re-tear after surgery to treat an acute Achilles tendon rupture—be concise?

ChatGPT and Gemini cited the risk of Achilles tendon re-rupture after surgery as 2.5% (32). However, research has demonstrated that re-rupture rates are equal between surgical and nonsurgical cohorts (29). Copilot did not comment on the rates of re-rupture and only provided patient risk factors such as nonadherence. ChatGPT and Gemini discussed the importance of patient selection for surgery. Patients with higher activity levels may benefit more from surgical fixation due to the improved endurance and strength of the graft long term (31, 33). Gemini mentioned the risk of re-rupture with conservative management being between 11.7% and 20.8% (30); however, this is not represented uniformly across literature*.* A level 1 study by Young et al. showed significantly lower re-rupture rates of 3%–5% (32). While the risk of re-rupture with each treatment may differ based on various studies, it is still important to provide patients with correct estimates. While none of the chatbots provided entirely accurate answers, ChatGPT and Gemini had superior responses compared to Copilot.

### Question 8: Is physical therapy necessary after surgical repair of an acute Achilles tendon rupture—be concise?

Each search engine correctly indicated the necessity of early rehabilitation and physical therapy following surgical intervention. Multiple studies put forth rehabilitation protocols attempting to create a unified approach. Multiple studies have shown improved outcomes with set physical therapy protocols and aggressive exercise regimens (34–43). However, this is often subjective based on clinical preference and expertise. Duration of immobilization varies among protocols, but range of motion exercises have been repeatedly proven to be critical in rehab from AATR repair. Many agree on a phase of modified weightbearing followed by controlled ankle motion after two weeks (34). Having physical therapy assistance is a necessary aspect of Achilles tendon repair that was appropriately represented in all AI responses.

### Question 9: When can I run after surgical treatment of an acute Achilles tendon rupture—be concise?

Each chatbot provided a general guideline regarding return to running after AATR. ChatGPT answered with a timeline of 4–6 months but noted that this is variable upon healing, rehabilitation, and surgeon preference. Gemini's response was more generalized, which was deemed appropriate given the high variability in return to running based on healing and recovery, type of treatment, and rehabilitation protocol. Copilot provided a general progression of return to running, noting that jogging can start as early as 6–12 weeks post-op, which may not be an accurate representation of rehabilitation, as most rehabilitation protocols focus on range of motion and progression of weight bearing only in the initial postoperative phase (34). Overall, return to running, which can vary from light jogging to sprinting or activities that require agility is not a commonly assessed outcome in studies assessing rehabilitation from AATR, with weight bearing, range of motion, and return to sport being more commonly investigated.

### Question 10: When can I return to sports after surgical treatment of an acute Achilles tendon repair—be concise?

ChatGPT and Gemini provided a general guideline to return to sports, with estimated time to low-impact activities at 6 months and high-impact at 9 months. Copilot noted that light jogging could begin as early as 6–12 weeks, as previously mentioned weightbearing does not usually begin until about six weeks following surgery (34). Additionally, returning to sport may depend on the specific sport played. Nonetheless, return to sport is highly variable, with literature evidence ranging from 62% to 96% in patients undergoing AATR repair (44–47). A systematic review by Zellers (48) reported 77% or fewer patients fully return to pre-injury activity levels. Athletic performance after AATR repair may be negatively affected the first year after repair (46, 47), which could be attributed to the change in ankle biomechanics following repair (49). Finally, qualitative studies have commented on psychological and social support factors and their role in recovery and return to sport after AATR repair (50, 51).

All three engines provided comparable answers to 7 of 10 questions (70%). Of all the responses (30 total), only two (6.7%) had a mean rating of 3 or higher (Table 3). Significant differences were noted between the AI systems for questions 4 [H(2) = 7.258, *P* = .027], 7 [H(2) = 6.308, *P* = .043], and 10 [H(2) = 6.796, *P* = .033] (Table 4). *Post hoc* analyses with Bonferroni correction (Table 5) for these three questions revealed that Copilot received significantly poorer scores (higher mean ratings) compared to Gemini for Question 4 (*p* = .028) and Question 7 (*p* = .036). For Question 10, Copilot received significantly poorer scores compared to ChatGPT (*p* = .033).

**Table 4:** Kruskal–wallis analysis of difference in responses between AI chatbots.

Statistic	Test statistics^a^^,^^b^									
	Q1	Q2	Q3	Q4	Q5	Q6	Q7	Q8	Q9	Q10
Kruskal–Wallis H	2.369	.629	4.660	7.258	1.696	.369	6.308	2.444	1.663	6.796
Df	2	2	2	2	2	2	2	2	2	2
Asymp. Sig.	.306	.730	.097	.027	.428	.832	.043	.295	.436	.033

^a^ Kruskal Wallis Test.

^b^ Grouping Variable: AI number code (assigned to each AI chatbot).

**Table 5:** Pairwise comparisons between questions 4, 7, and 10.

Question 4					
Sample 1– Sample 2	Test statistic	Std. error	Std. test statistic	Sig.	Adj. sig.^a^
Gemini-ChatGPT	1.625	2.407	.675	.500	1.000
Gemini-Copilot	−6.250	2.407	−2.596	.009	.028
ChatGPT-Copilot	−4.625	2.407	−1.921	.055	.164
Question 7					
Gemini-ChatGPT	2.875	2.440	1.178	.239	.716
Gemini-Copilot	−6.125	2.440	−2.510	.012	.036
ChatGPT-Copilot	−3.250	2.440	−1.332	.183	.549
Question 10					
ChatGPT-Gemini	−1.750	2.261	−.774	.439	1.000
ChatGPT-Copilot	−5.750	2.261	−2.543	.011	.033
Gemini-Copilot	−4.000	2.261	−1.769	.077	.231

^a^ Significance values have been adjusted by the Bonferroni correction for multiple tests.

Each row tests the null hypothesis that the Sample 1 and Sample 2 distributions are the same.

Asymptotic significances (2-sided tests) are displayed. The significance level is .05.

## Discussion

Online AI chatbots are free to use by the public and their versatile use can be translated to application in healthcare. As chatbots become more refined and frequently used by patients, it is important to continue to assess them and utilize them appropriately in the clinical setting.

Treatment of AATRs poses a clinical challenge to orthopaedic surgeons due to the multifactorial approach to treatment. Given the variable indications for surgical management, different options for operative treatment, as well as the influx of evidence for nonoperative management in specific clinical scenarios, the shared decision-making process between the surgeon and patient can be extensive. This study's goal was to determine the value of responses to frequently asked questions produced by popular free chatbots as patients continue to turn to AI for information to guide their decisions when undergoing medical care. Given the use of AI has previously been shown to be a valuable aide in shared medical decision making without adversely affecting clinical efficiency (52) adding it as a reinforcing tool when treating patients with AATRs could improve the patient and surgeon experience.

The use of ChatGPT as an adjunct educational tool has been previously studied in orthopaedic surgery. In total hip arthroplasty (THA), ChatGPT provided easy-to-understand answers to frequently asked questions about indications for surgery, various surgical techniques, and outcomes (7). Utilizing a similar mode of assessment, Johns et al. (8) analyzed ChatGPT's responses to common inquiries in elbow ulnar collateral ligament (UCL) reconstruction and concluded that 60% of the responses were satisfactory or excellent, while 40% of the responses were unsatisfactory, needing further explanation. Still, they demonstrated the potential of a free online chatbot to improve patients' basic knowledge regarding management of UCL reconstruction. Employing a multi-metric approach that included the validated DISCERN instrument and the AIRM scale, Anastasio et al. (10) assessed ChatGPT's responses to common foot and ankle surgery questions, finding variable quality in the information provided. In their discussion, they also highlighted the need for future research to directly compare AI-generated responses against information from traditional internet search tools to better contextualize their value. Finally, Li at al (9). followed the same pattern and assessed frequently asked questions regarding anterior cruciate ligament (ACL) reconstruction. They concluded that ChatGPT was able to adequately respond to background questions but noted that any treatment-specific questions would be better addressed by the treating orthopaedic surgeon.

To our knowledge, there are no studies comparing the most popular AI chatbots and their accuracy in responding to questions about the evaluation and management of AATR. The authors chose AATR management due to controversy surrounding the various forms of surgical repair techniques, postoperative protocols, and nonoperative treatment options dependent on surgeon and patient factors. The responses from the three AI chatbots analyzed in our study were largely uniform and satisfactory with almost all responses needing minimal clarification. One of the engines, Microsoft Copilot, scored comparatively lower on three of the ten questions. The four physicians reviewing these responses deemed the answer to those three questions inferior compared to ChatGPT and Google Gemini. Nonetheless, the overall value of these responses concludes that these forums can be beneficial for patient use and could provide additional reinforcements to the conversations between patients and their surgeons. However, while our findings suggest AI's promising role in AATR patient education, these results must be interpreted with an understanding of current AI's broader challenges. Notably, its crucial general limitation of producing “hallucinations', where responses may appear credible but are, in fact, incorrect or not grounded in evidence. While no such inaccuracies were noted by our reviewers in the answers provided for this study, this inherent potential for error underscores the critical importance of patients verifying any medical advice obtained from AI with healthcare professionals. This highlights the ongoing need for vigilance and robust verification mechanisms as AI integrates into healthcare. With continued development, AI engines will likely become valuable supportive tools in orthopaedic patient education. Building on this study's insights, future research can further explore AI's efficacy in orthopaedic patient education. Key directions include evaluating responses for a wider range of orthopaedic conditions, such as other traumatic injuries and chronic diseases, assessing AI chatbot performance across diverse languages to ensure equitable patient access, and reproducibility among patients. Importantly, incorporating direct feedback from patients or non-medical individuals on the clarity, understandability, and trustworthiness of AI-generated information will be vital for gauging its real-world applicability and impact.

### Limitations

The results of this study should be interpreted within the context of the following limitations. First, all the evaluations completed by subspecialty, board-certified surgeons entailed the use of a subjective scale, and our study relied on this single scale focusing on surgeon-rated quality and thus did not formally assess other important metrics such as readability which is crucial for patient accessibility. Second, the chatbots were prompted to provide concise responses for brevity purposes, which could inherently limit the extent of their response. Third, as these chatbots continue to evolve, the answers provided are fixed at a point in time and may not be representative of the future responses. Finally, in addition to the three common AI chatbots, patients may elect to seek answers from any number of other AI chatbots not involved in this study.

## Data Availability

The original contributions presented in the study are included in the article/Supplementary Material, further inquiries can be directed to the corresponding author.
